# The association between conjunctival and scleral thickness and ocular surface ultraviolet autofluorescence

**DOI:** 10.1038/s41598-023-35062-2

**Published:** 2023-05-16

**Authors:** Pryntha Rajasingam, Alyra Shaw, Brett Davis, David Alonso-Caneiro, Jared Hamwood, Michael Collins

**Affiliations:** Contact Lens and Visual Optics Laboratory, School of Optometry and Vision Science, Queensland University of Technology, Victoria Park Road, Kelvin Grove, QLD 4059 Australia

**Keywords:** Predictive markers, Tomography

## Abstract

Ultraviolet autofluorescence (UVAF) imaging is used to visualise ocular surface changes due to sunlight exposure and so is considered to be a biomarker for UV damage. The conjunctival and scleral thicknesses of participants with and without ocular surface UVAF were measured to examine the UVAF associated tissue thicknesses. The presence of UVAF on the ocular surface was associated with significant differences in tissue thickness including thinner conjunctival epitheliums and thicker scleras but predominantly thickening of the conjunctival stroma. Participants were also classified into four groups according to the presence and absence of UVAF on both the temporal and nasal conjunctivas. It was noted that for those that had only nasal UVAF, the temporal conjunctival stroma was significantly thicker even without the presence of UVAF. Some participants with temporal UVAF had signs of pinguecula observed with slit lamp examination and some had OCT SLO enface imaging darkening. These findings highlight the potential of techniques other than slit lamp examination, including tissue thickness measurement and UVAF photography, in the detection of early UV-related changes to the ocular surface.

## Introduction

Fluorescence occurs when a substance is excited by radiation of a certain wavelength (often ultraviolet) and re-emits radiation usually of a longer wavelength (often visible light). Using instrumentation based on the Wood’s light principle used in dermatology, Ooi, Coroneo and colleagues described the phenomenon of ocular surface ultraviolet autofluorescence (UVAF) associated with pterygium and found that it was correlated with ocular sun exposure^1^. An increased prevalence of pterygium has been found to be associated with a larger UVAF area^2–5^. Absorption of UV radiation is thought to cause damage to ocular structures at the cellular level, which results in autofluorescence^6,7^. There are many possible changes to the conjunctiva that have been suggested to result from UV radiation including changes to collagen, elastin, lysosomes, mitochondria, cytokines, NADH, tryptophan, lipofuscin or matrix metalloproteinases, but the exact mechanism of conjunctival autofluorescence is not known^8^.

There are potential implications of UVAF not only for ophthalmohelioses (sun-related ocular diseases) including pterygium, cataract and squamous cell carcinomas of the cornea and conjunctiva, but also skin conditions such as sunburn, solar keratosis, basal and squamous cell carcinomas, and malignant melanomas. However, UVAF occurs not only with visible UV-related changes to the ocular surface such as pterygium and pinguecula, but also when there are not any ocular surface changes visible with white light and so it has been suggested that UVAF may be a precursor to clinical manifestations of damage. In these instances (without the presence of pterygia), the UVAF area has also been shown to be positively correlated to the time spent outdoors^3,5,9–12^. Consequently, UVAF may be a biomarker for ocular sun exposure and an indicator of ocular surface pathology risk^5^.

UVAF may also be an important tool in myopia research, as outdoor light exposure has also been associated with slower myopia progression^13–15^. Several researchers have reported that UVAF area is inversely associated with myopia, with larger UVAF areas associated with less or no myopia^16–19^ and also associated with the degree of myopia^17^.

UVAF provides an objective measure of previous outdoor light exposure, rather than subjective questionnaires that rely on a participant’s recall ability^2,20^. Despite showing potential as a biomarker, it remains unclear what the UVAF specifically represents and the timeframe over which the UVAF develops. It has been suggested that UVAF may be due to cumulative medium-term sun exposure over the preceding months^16^ or related to lifetime sun exposure^11^. While histologically pinguecula are characterized by elastotic degeneration of collagen with hyalinized connective tissue^21–23^, to our knowledge the conjunctival and scleral thicknesses associated with UVAF have not previously been considered. This study aims to compare the conjunctival and scleral tissue thicknesses in individuals with and without UVAF and to also study corresponding UVAF images, SLO enface images and slit lamp examination.

## Methods

### Participants

This study was conducted according to the tenets of the Declaration of Helsinki including written informed consent with ethics approval from the UHREC. A total of 53 participants were enrolled in this study, however 9 were excluded due to poor fixation during the OCT measurement, as it required maintaining steady peripheral fixation for approximately 3 min. A further 2 participants were excluded due to conjunctival pigmentation which interfered with the UVAF photography and potentially OCT tissue thickness measurements. The remaining 42 participants were aged between 19 and 43 years of age (mean 28 ± 6 years), 22 were female and there were 25 Caucasian, 15 Asian and 2 participants of other ethnic backgrounds. Prior to the study, all participants underwent a screening examination to assess their ocular health, including slit lamp examination to record the presence of pinguecula (defined by both color and elevation), visual acuity and completion of a lifetime sun exposure questionnaire^24^. All participants had no significant history of ocular surgery or trauma. No participants wore rigid contact lenses and soft contact lens wearers discontinued contact lens wear 24 h prior to commencing the study to allow any substantial effect of contact lens wear on conjunctival or scleral morphology to resolve.

### Ultraviolet autofluorescence (UVAF) photography

The right eye nasal and temporal UVAF photographs were taken with a custom-built camera system using a Nikon D90 digital camera with ISO speed 2,000 and exposure time of 1/20 s. The UVAF system was connected to a laptop computer (qDSLRDashboard V3.5.3 software) to provide a magnified live view of the eye during measurement. Each participant’s eye was first illuminated with white (incandescent) light to obtain control images (aperture size was set to f/14) and was then illuminated with 375 nm ultraviolet light (full width at half maximum of 13.6 nm) to obtain fluorescence images (aperture size was set to f/4.8). During this procedure, the participant was directed to fixate at an external target (red LED light) at 27° off-axis nasally and temporally. Three images of the nasal and temporal conjunctivas of the right eye were captured for each light source. The images were saved in RAW file format (.NEF) which allowed uncompressed and unprocessed images to be analysed. At the end of the procedure, an image of a millimetre ruler was captured to provide a scale reference for subsequent analysis. Based on the UVAF photographs, the participants were categorised according to the presence of UVAF (n = 25) or absence of UVAF (n = 17) on the temporal conjunctiva. For further analysis, the participants were split into groups based on the presence of UVAF in both nasal and temporal regions resulting in 4 subgroups (Table 1).

**Table 1:** Participants classified into 4 subgroups depending on the presence of UVAF in the nasal and temporal conjunctivas.

Subgroup	Temporal UVAF	Nasal UVAF	UVAF location (right eye)
None (n = 10)	No	No	
Nasal (only) (n = 7)	No	Yes	
Temporal (only) (n = 8)	Yes	No	
Both nasal and temporal (n = 17)	Yes	Yes	

### Anterior segment optical coherence tomography (AS-OCT)

**Figure 1:**
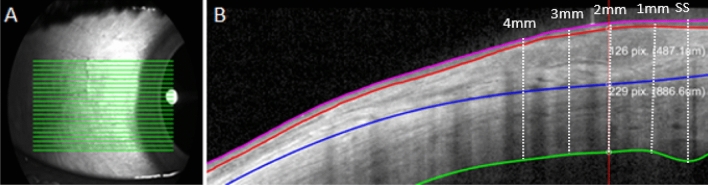
Anterior eye OCT imaging volume scan (21 lines) captured (**A**), segmentation export of 1 line scan showing the manually segmented image with the 3 tissue layers analysed: conjunctival epithelium (pink to red lines), conjunctival stroma (red to blue lines) and stroma (blue to green lines) (**B**). This was completed for each of the 21 scan lines shown in A and from the scleral spur (SS) at 0 mm through to 4 mm.

The temporal conjunctival and scleral thickness of the right eye of each participant were measured using the Heidelberg Spectralis OCT (Heidelberg Engineering, Heidelberg, Germany) with anterior segment imaging module. The device captures cross-sectional images at a scanning speed of 40,000 A scans/second, axial resolution of 3.9 µm and transverse resolution of 14 µm using a central wavelength of 870 nm. Two scans were taken consisting of 21 horizontal lines, with line number 11 of the 21 horizontal line scans (i.e. the central line) positioned close to the lower edge of the bright corneal reflection in the SLO enface image to ensure a consistent scanning location for each participant (Fig. 1A). The scan pattern covered a 11.1 × 5.6 mm area and the automatic real time eye tracking (ART, 30 images averaged) was also used to improve image quality with an average quality score of 32 ± 6 dB. The instrument’s scleral imaging mode with enhance depth imaging (EDI) was activated to obtain a high-resolution cross-sectional image of the conjunctiva and sclera. To obtain the best alignment for the temporal conjunctiva scans, the participants fixated using an external fixation target (a green LED) placed at a distance of 2 m from the eye at an angle of 40° nasal. An enface image, using the instrument’s scanning laser ophthalmoscope (SLO), was also captured with a 27° fixation to correspond to the UVAF photographs. A photo of a millimetre ruler was also captured to provide a scale reference for the analysis.

### Data analysis

The OCT images were exported and analysed with a semi-automated procedure using custom written software, where any errors in the automatic segmentation were manually corrected and the location of the scleral spur was marked using the ciliary muscle landmark method^25^. This semi-automated method was used to segment the anterior and posterior boundaries of the corneal epithelium and for the posterior boundary of the sclera. The episcleral boundary between the conjunctival stroma and sclera was manually segmented by marking the posterior edge of the episcleral blood vessels. Data from the two repeated scans were averaged for conjunctival epithelium, conjunctival stroma, and scleral thickness for all 21 lines from each OCT image. Using the scleral spur as the starting location, thicknesses were compared at the scleral spur 0 mm, 0–1 mm, 1–2 mm, 2–3 mm and 3–4 mm (measured along the arc of the curved posterior scleral boundary) according to the method of Read et al.^25^ (Fig. 1B). This method has previously been reported to be highly repeatable for conjunctival and scleral thickness data, with intraclass correlation coefficients greater than 0.95. Average thickness maps (pseudo-coloured) were also generated and plotted on an SLO enface image for visualisation of the approximate location of the tissue thicknesses. For the analysis of the four subgroups, the group with no nasal or temporal UVAF were used as the reference to calculate the difference in tissue thickness compared to those with nasal UVAF, temporal UVAF and both nasal and temporal UVAF.

**Figure 2:**
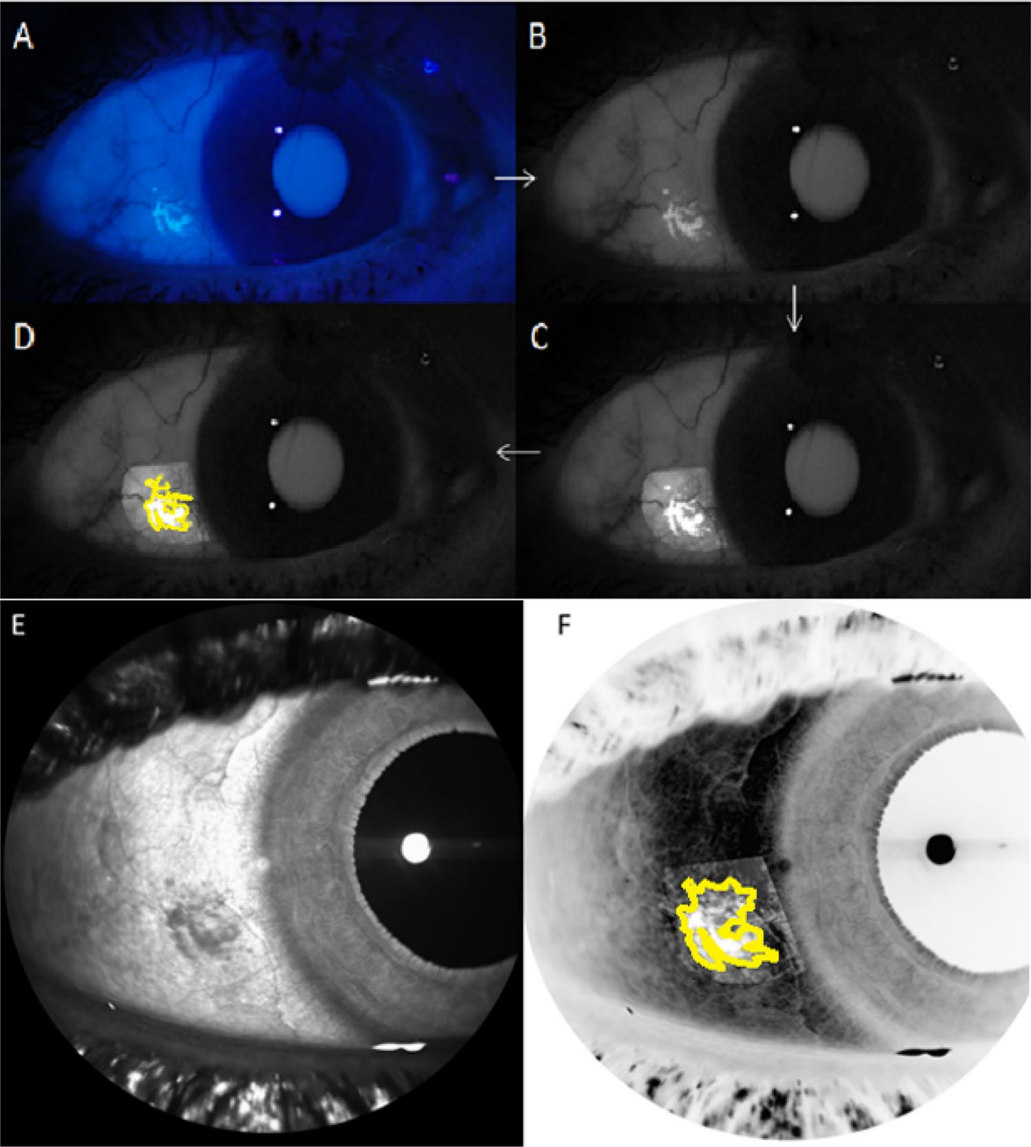
Original UVAF photograph (**A**), green channel image (**B**), processed image (**C**), analysed image with UVAF outline (**D**), corresponding OCT SLO enface image (**E**) and analysed OCT SLO enface image with outline (**F**).

The UVAF photographs (Fig. 2A) were analysed by extracting the green channel of the RAW photograph (Fig. 2B) and processed in customised software to normalise the intensity across the image (Fig. 2C). The processed image was then imported to ImageJ to use the freehand tool to trace around the edges of the UVAF region to calculate the UVAF area (Fig. 2D). The dark patch in an SLO enface image was also mapped using a similar method (removing the tilt and taking the negative image) so that the edges of the region could be traced to calculate an SLO enface area (Fig. 2 E and F).

### Statistical analysis

All data were entered into an Excel file (Microsoft Inc, Redmond, WA, USA) and transferred to SPSS (version 26, IBM, Armonk, New York, USA) for analysis. Tissue thicknesses for all lines and locations were approximately normally distributed and it was considered valid to apply parametric tests.

To examine the thickness data, a repeated measures MANOVA was used for all tissue thicknesses, with two within subject factors: scan line (1 through to 21) and measurement location with respect to the scleral spur (0 mm, 0–1 mm, 1–2 mm, 2–3 mm, 3–4 mm) and one between-subject factor (with or without UVAF groups). A second repeated measures MANOVA was conducted to analyse the tissue thicknesses with the between-subjects factor dividing the participants into 4 subgroups determined by UVAF presence in both the nasal and/or temporal conjunctivas (Table 1). A MANOVA was also used to analyse the UVAF group OCT tissue thickness with a between-subjects factor splitting the participants into two groups determined by the presence and absence of pinguecula diagnosis from slit lamp examination. A further MANOVA analysed the UVAF group OCT tissue thicknesses with the between-subjects factor splitting the participants into the two groups determined by the presence and absence of SLO enface dark region. Post hoc analysis with multiple comparison correction was completed for any factors with more than two levels. Correlations between the UVAF area and tissue thicknesses were explored with Pearson correlations for each OCT scan line and location from the scleral spur. Other correlations with tissue thicknesses investigated the effect of gender, habitual spectacle wear and lifetime sun exposure questionnaire score. Pearson correlation also investigated an associated between the UVAF area and SLO enface dark region area. For all analyses, p-values < 0.05 were considered to be statistically significant.

## Results

**Figure 3:**
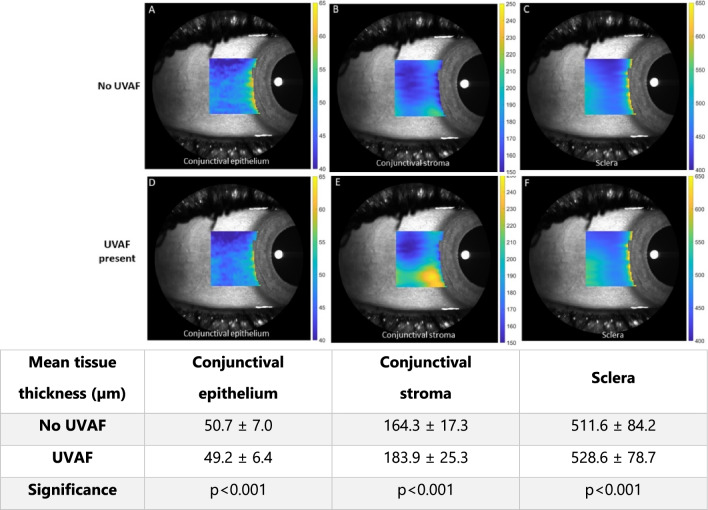
Mean thickness maps for No UVAF (**A** to **C**) and UVAF groups (**D** to **F**) overlayed on an SLO enface image to show approximate position. Group mean (± SD) tissue thicknesses (conjunctival epithelium, conjunctival stroma and scleral) for both groups in the adjoining table.

**Figure 4:**
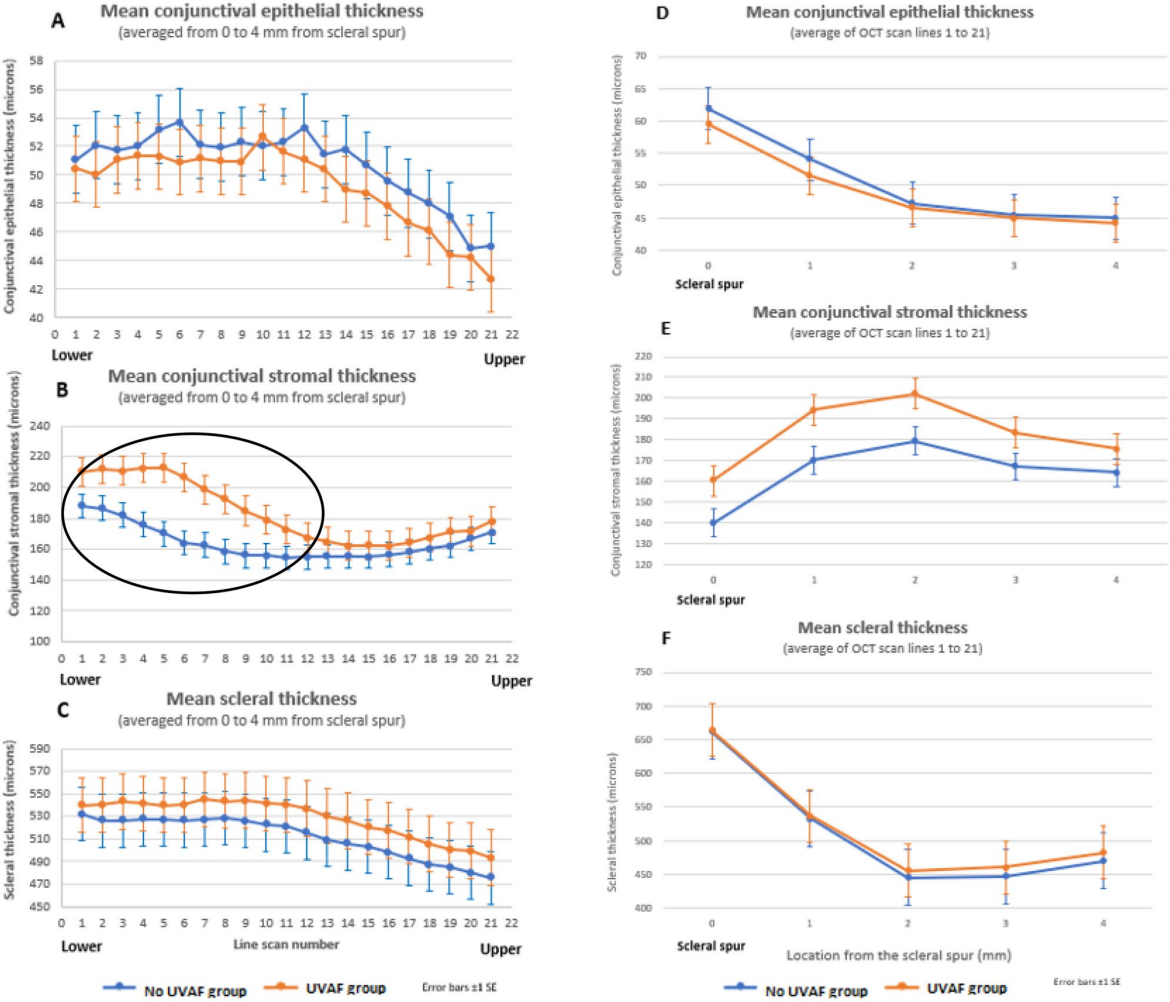
Mean conjunctival epithelium, conjunctival stroma, and scleral thickness for the no UVAF group (blue) and UVAF group (orange) averaged for each scan line (**A** to **C**) and location from the scleral spur (**D** to **F**). Circle indicates the localised difference between the two groups for scan lines 1 to 11 in the mean conjunctival stromal thickness (**B**). Error bars ± 1 SE.

Significant differences in tissue thickness were observed for the UVAF group compared to the no UVAF group for all three tissue layers: conjunctival epithelium, conjunctival stroma, and sclera (MANOVA, all p < 0.001). Those with UVAF had thinner conjunctival epitheliums, thicker conjunctival stromas and thicker scleras (Fig. 3). The conjunctival epithelium and sclera showed similar thickness differences between the two groups for nearly all OCT scan lines (vertical position) and locations (horizontal position), however the conjunctival stroma had a localised region with a larger difference between the two groups, corresponding to the approximate location of the UVAF (lines 1 to 11, lower half of the OCT volume scan) (Fig. 4 A to C). Considering the distance away from the scleral spur, the averages for the two groups show decreasing conjunctival epithelial thickness, increasing conjunctival stromal thickness and decreasing stromal thickness for both the no UVAF and UVAF groups (Fig. 4 D to F). The age of the participants of the two groups was not statistically significant (T-test, p > 0.1) with mean ± SD of 26.2 ± 4.4 and 28.8 ± 6.4 years for the no UVAF and UVAF groups, respectively. There was also no significant difference in gender between the two groups (Chi-square, p > 0.1) with 41% and 52% being male in the no UVAF and UVAF groups, respectively.

**Figure 5:**
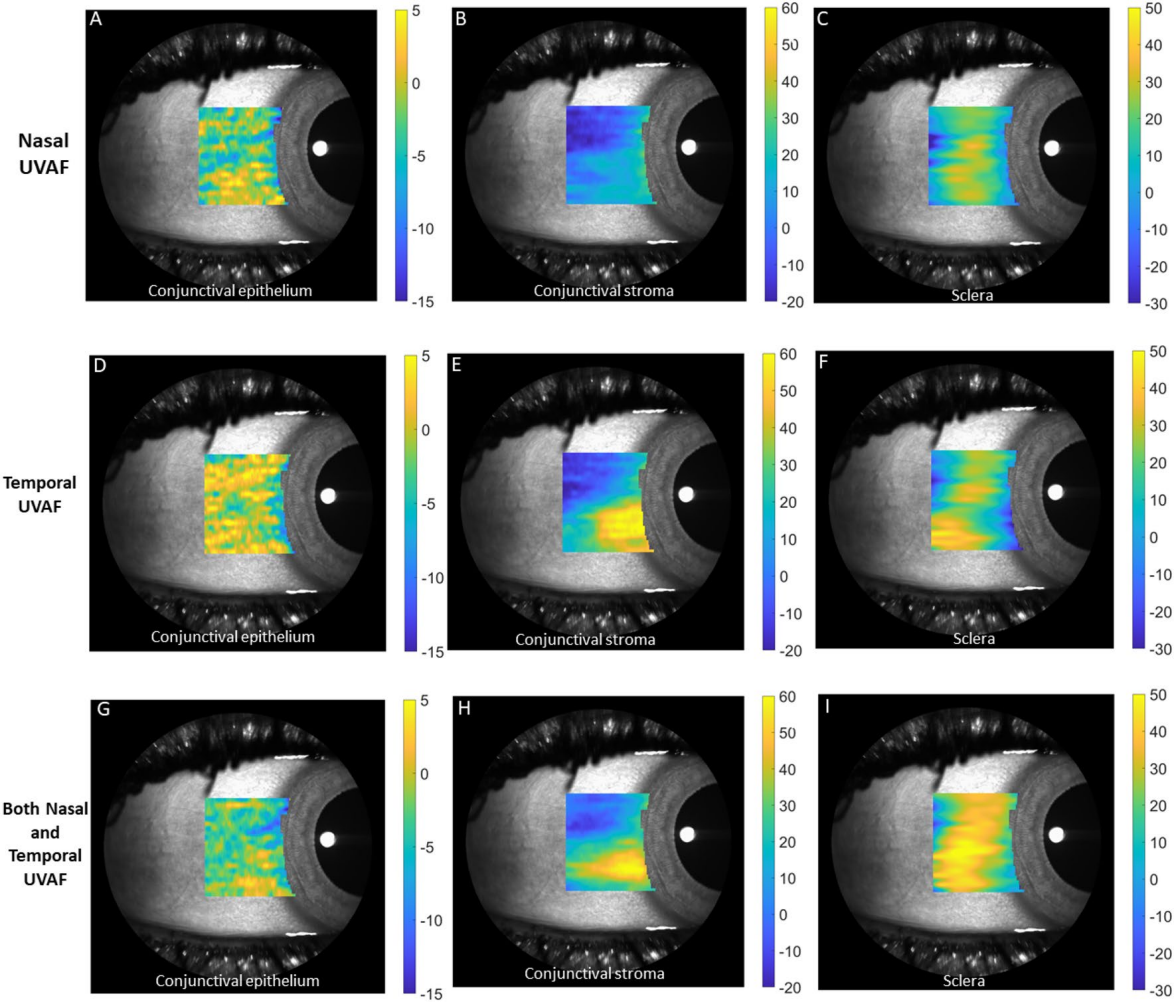
Mean thickness difference maps for the groups Nasal only UVAF (**A** to **C**), Temporal only UVAF (**D** to **F**) and Both Nasal and Temporal UVAF (**G** to **I**) compared to the No UVAF group overlayed on an SLO enface image. Note the vertical scales were generated for each type of tissue and so the value of 0 (no difference) is not represented by the same color for each tissue.

**Figure 6:**
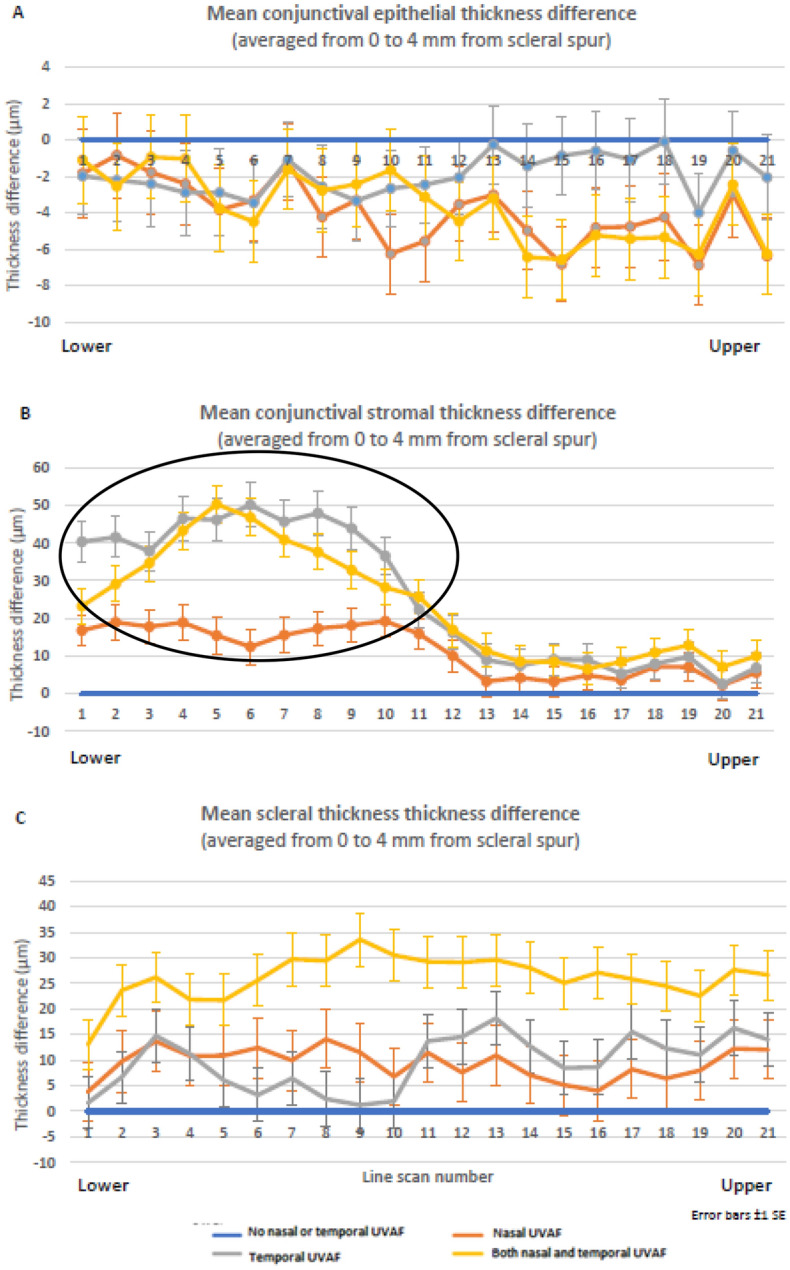
Mean difference in conjunctival epithelium (**A**), conjunctival stromal (**B**) and scleral (**C**) thickness for each of the four subgroups compared to no nasal or temporal UVAF group for each scan line. Circle indicates the greatest localised difference between the groups for scan lines 1 to 11 (B). Error bars are ± 1 SE.

Further analysis was conducted with the participants split into four subgroups depending on UVAF presence of both temporal and nasal UVAF (refer to Table 1). Due to anatomical tissue thickness variations depending on location, this data was analysed as the difference compared to the group without nasal or temporal UVAF. Mean thickness difference maps (conjunctival epithelium, conjunctival stroma, and sclera) for the 3 groups (compared to the group without nasal or temporal UVAF) are shown in Fig. 5. Compared to the no UVAF group, all the other groups had thicker temporal conjunctival stromas, particularly for those with only temporal UVAF, but also for only nasal UVAF group (MANOVA posthoc analysis all p < 0.001, Fig. 6B). Similar to the two-group analysis (UVAF versus no UVAF groups), the conjunctival epithelium was consistently thinner for all scan lines (MANOVA, all p < 0.001) and sclera was consistently thicker compared to the group with neither temporal or nasal UVAF (Fig. 6A and C). The mean and standard deviation of tissue thickness differences for those with only nasal UVAF, only temporal UVAF and both nasal and temporal UVAF, compared to those without nasal or temporal UVAF, are presented in Table 2.

**Table 2:** Group mean (± SD) tissue thicknesses (conjunctival epithelium, conjunctival stroma and scleral) for those with nasal UVAF, temporal UVAF and both nasal and temporal UVAF, compared to those without nasal or temporal UVAF.

Temporal tissue thickness (µm)	Conjunctival epithelium	Conjunctival stroma	Sclera
Nasal only UVAF group	−3.9 ± 4.8	11.4 ± 4.3	9.4 ± 5.8
Temporal only UVAF group	−1.9 ± 4.9	25.8 ± 4.9	9.6 ± 5.3
Both nasal and temporal UVAF group	−3.7 ± 5.2	23.6 ± 4.5	26.2 ± 5.0

The mean UVAF area (for the UVAF group only, n = 25) was 10.8 ± 6.4 mm^2^ with a median of 9.2 mm^2^, ranging from 2.5 to 25.3 mm^2^.

All participants that had a pinguecula on the temporal conjunctiva also had UVAF in this region (n = 12). However not all participants with UVAF were classified as having pingueculas from slit lamp examination (n = 13). Examining the OCT tissues thickness for these two groups, those with visible pinguecula signs had statistically thinner conjunctival epitheliums (p < 0.001) and thicker stromas (p < 0.001) for all scan lines and location with respect to the scleral spur. Interestingly, the group with pinguecula had thinner conjunctival stromal thicknesses overall, however on closer examination the scan lines outside the region of the pinguecula had thinner conjunctival stromas while scan lines 3 through 7 (corresponding to the pinguecula location) had thicker conjunctival stromas.

When capturing the OCT images it was noted that some individuals had a darker region corresponding to the location of UVAF (for example Fig. 2E). The UVAF group (n = 25) was split into those with SLO enface darkening (n = 13) and those without SLO enface darkening (n = 12) and comparisons were made between these groups. The corresponding UVAF area was not statistically different between these two groups, 11.0 ± 5.2 and 10.5 ± 7.7 mm^2^ respectively (T test, p > 0.05). For those with an SLO enface darkening, the area of this region was significantly positively correlated to the UVAF area (r = 0.82, p < 0.001), with the SLO enface darker area being smaller than the corresponding UVAF area. The participants with an SLO enface darkening had statistically significant differences in tissue thickness with thicker conjunctival epitheliums (p < 0.001), thicker conjunctival stromas (p = 0.008) and thicker scleras (p < 0.001) compared to those with UVAF but without SLO enface darkening. There were no significant correlations between OCT tissue thicknesses with gender, habitual spectacle wear, and lifetime sun exposure questionnaire score (all p > 0.05).

## Discussion

This study reports the in vivo thickness of the conjunctiva and sclera using spectral domain AS-OCT and demonstrates significant differences in these tissues with and without the presence of UVAF. To our knowledge this is the first study considering ocular surface tissue thickness changes associated with UVAF. With the presence of UVAF a localised, thicker conjunctival stroma was observed. Similarly, it is known that pingueculas are associated with an increased conjunctival stromal thickness with the cellular changes being hyalinised subepithelial collagen and elastotic degeneration^21–23^. In addition, a slightly thinner conjunctival epithelium was observed. Thinner conjunctival epitheliums have been previously reported associated with a decline or suppression of metabolic function^26^ and a similar mechanism may be involved with UVAF associated tissue changes. Specifically for pinguecula, any epithelial changes may include atrophy, hyperplasia, metaplasia or dysplasia^21,27^. It should be noted that the conjunctival epithelial difference between the UVAF and no UVAF groups was only 1.5 microns. Although the UVAF group had consistently thinner conjunctival epitheliums vertically and horizontally, this small difference may not be clinically significant. Along with the conjunctival epithelial and stromal changes with UVAF, the sclera was also thicker, which to our knowledge has not been previously reported.

Variations in tissue thickness occurred for each of the horizontal line scans and along each of these scans away from the scleral spur. The pattern of tissue thickness for all participant groups considered in this study was consistent with previous reports where the conjunctiva is thinnest close to the scleral spur and thickens away from the scleral spur while the scleral thickness was opposite, being thickest at the scleral spur and reducing with distance away from this point^28,29^. Due to the large variation in thickness depending on the measurement location, it can be difficult to compare between studies. However, the average tissue thickness (conjunctival epithelium, stroma and sclera) for the no UVAF group in this study showed reasonable agreement with previous reports^3,26,28,30,31^. Changes to tissue thickness can occur with age^25,26,32,33^ and males on average have thicker conjunctivas and scleras than females^25,34–37^. These factors are thought to have minimal influence in this study as there were no significant differences between the participant groups when analysed for age or gender.

While pterygia are more likely to affect the nasal conjunctiva^38^, pinguecula are reported as having a similar prevalence on the nasal and temporal sides^39,40^. There is conjecture regarding the location of UVAF, with some reporting greater nasal prevalence^11,12,41^, while equal nasal and temporal prevalence has also been reported^1^. Similarly, regarding the UVAF area, the nasal conjunctiva has been reported to have greater UVAF changes than temporal^10,12,41,42^, though no statistical difference between nasal and temporal UVAF areas has also been reported^10,11,43^. In this study considering the four subgroups determined by the presence of nasal and temporal UVAF, there were approximately equal proportions as there were seven participants with only nasal UVAF and eight participants with only temporal UVAF.

An interesting finding was that the temporal conjunctival stromas of those with only nasal UVAF, while not as thick as those with temporal UVAF, were statistically thicker than the participants without either nasal or temporal UVAF. So, for eyes with UVAF on the contralateral nasal conjunctiva, there were temporal conjunctival tissue thickness changes even though there was no temporal UVAF. In a similar way, preclinical conjunctival changes due to ultraviolet light damage have been measured with confocal microscopy for eyes that were clinically normal when the fellow eye had a pterygium or pinguecula^44^. Although nasal OCT data was collected for this study, due to the considerable time involved in OCT segmentation and analysis, only the temporal conjunctival scans for each participant were analysed. It is acknowledged that analysis of the corresponding nasal data would provide further insight into the tissue changes associated with ocular surface UVAF and may be possible in the future with further development of automatic segmentation techniques. It is also acknowledged that dividing the participants into 4 subgroups resulted in some small group sizes (n = 10, 7, 8 and 17). Additionally, for the conjunctival epithelium and sclera, changes were small. Although significant differences in tissue thickness were found in the analysis, this should be investigated with larger sample sizes for confirmation.

The area of UVAF has been considered by many studies, however there is large variation in results depending on the age and geographic location of the participants included. The results of this study (UVAF median = 9.2 mm^2^ and mean = 10.8 ± 6.4 mm^2^) are comparable to other Australian or Norfolk Island based studies^11,18–20,45^. Some studies report much smaller UVAF areas, less than 3 mm^2^, when conducted in Europe^9,10,16,42^ or when the participants were children^12,17^.

About half of the participants with UVAF had clinical signs (change in tissue colour and thickness) of a pinguecula from slit lamp examination, while the other half had no clinical signs of a pinguecula. Similarly one study recorded 30% of children with UVAF to have signs of a pinguecula^1^. In this study, the participants with pinguecula had greater thickness changes than those without pinguecula, with thinner conjunctival epitheliums, thicker conjunctival stromas and thicker scleras. While not considered in this study, it has been reported that the UVAF area is larger than the visible pinguecula area from slit lamp examination for 71.4% of lesions^41^ with the remainder of eyes having equal UVAF and visible pinguecula areas.

The SLO enface darkening was an unexpected finding occurring for approximately half of the participants with UVAF, particularly as UVAF photography uses an excitatation wavelength of 375 nm while the Spectralis OCT uses 820 nm during SLO enface imaging. Some possible explanations include that the OCT is highlighting different cellular changes in comparison to UVAF photography, or that the increased tissue thickness also affects its composition increasing the scattering of light.

There are some limitations of this study that should be considered. For consistency, the OCT volume scans were aligned for each individual with scan line 11 at the inferior edge of the corneal reflex, as the positioning of an OCT scan line with the corneal reflection has been shown to have a high repeatability^25^. All data was taken for same degree of eye turn and images analysed as captured. This means that thickness was not always measured perpendicular to the epithelium for all locations (0 to 4 mm). However this is considered to have little bias on the results as reanalysis of similar data using both ‘axial’ and ‘normal’ thickness measures found small mean differences of 0.6 µm and 1.3 µm for the scleral and conjunctiva respectively^25^. Also a correction for refractive distortion was applied to the OCT images and the measurement location was defined based on the arc of the curved posterior sclera for more robust between-subject comparisons^25^. The conjunctiva and sclera are known to have diurnal variations which may have impacted the results of this study. However the greatest changes are known to occur after waking^29^ and so the effect of diurnal variations were minimized in this study by taking measurements between 8 am and 5 pm and at least 2 h after waking. Without reliable landmarks the individual variability in the exact location of the UVAF region made it impossible to directly match the UVAF photographs to the OCT data. However, for the majority of individuals the UVAF region was below the mid-limbus which approximately corresponded to the corneal reflex and line number 11 of the OCT scan.

This study has confirmed that UVAF is a precursor to clinical slit lamp visibility of sunlight associated conjunctival changes. Furthermore, it was found that temporal conjunctival stromal thickness changes may occur even without temporal UVAF when the nasal conjunctival exhibits UVAF. This suggests that tissue thickness changes may precede UVAF. Additionally, a darkened region was evident on the SLO enface image for approximately half the individuals with UVAF. Based on these observations we propose that ocular surface UV-related changes would first be detectable by tissue thickness changes (measured by OCT), then by UVAF, and finally by clinical manifestations with slit lamp white light examination or by the detection of an SLO enface darkened region. These findings highlight the importance of techniques other than slit lamp examination, such as tissue thickness measurement and UVAF photography in the detection of early UV-related changes to the ocular surface.

## Data Availability

The data that support the findings of this study are available from the corresponding author (AS) upon reasonable request.

## References

[1] Ooi J-L (2006). Ultraviolet fluorescence photography to detect early sun damage in the eyes of school-aged children. Am. J. Ophthalmol..

[2] Lingham G (2019). Repurposing blue laser autofluorescence to measure ocular sun exposure. Clin. Experiment. Ophthalmol..

[3] McKnight CM (2015). Pterygium and conjunctival ultraviolet autofluorescence in young Australian adults: The Raine study. Clin. Experiment. Ophthalmol..

[4] Ooi J-L (2007). Ultraviolet Fluorescence Photography: Patterns in Established Pterygia. Am. J. Ophthalmol..

[5] Sherwin JC (2013). The association between pterygium and conjunctival ultraviolet autofluorescence: The Norfolk Island Eye Study. Acta Ophthalmol..

[6] Asawanonda P, Taylor CR (1999). Wood's light in dermatology. Int. J. Dermatol..

[7] Sandby-Møller J, Thieden E, Philipsen PA, Heydenreich J, Wulf HC (2004). Skin autofluorescence as a biological UVR dosimeter. Photodermatol. Photoimmunol. Photomed..

[8] Svistun E (2004). Vision enhancement system for detection of oral cavity neoplasia based on autofluorescence. Head Neck.

[9] Haworth KM, Chandler HL (2017). Seasonal effect on ocular sun exposure and conjunctival UV autofluorescence. Optom. Vis. Sci..

[10] Kearney S, O'Donoghue L, Pourshahidi LK, Richardson PM, Saunders KJ (2016). The use of conjunctival ultraviolet autofluorescence (CUVAF) as a biomarker of time spent outdoors. Ophthalmic Physiol. Opt..

[11] Sherwin JC (2011). Distribution of conjunctival ultraviolet autoflourescence in a population-based study: The Norfolk Island Eye Study. Eye (Lond.).

[12] Sun C (2017). Conjunctival ultraviolet autofluorescence as a measure of past sun exposure in children. Cancer Epidemiol. Biomark. Prev..

[13] Read SA, Collins MJ, Vincent SJ (2015). Light exposure and eye growth in childhood. Invest. Ophthalmol. Vis. Sci..

[14] Dirani M (2009). Outdoor activity and myopia in Singapore teenage children. Br. J. Ophthalmol..

[15] Rose KA (2008). Outdoor activity reduces the prevalence of myopia in children. Ophthalmology.

[16] Kearney S (2019). Conjunctival ultraviolet autofluorescence area, but not intensity, is associated with myopia. Clin. Exp. Optom..

[17] Kumar S (2021). Myopia, melatonin and conjunctival ultraviolet autofluorescence: A comparative cross-sectional study in Indian Myopes. Curr. Eye Res..

[18] McKnight CM (2014). Myopia in young adults is inversely related to an objective marker of ocular sun exposure: The Western Australian Raine Cohort Study. Am. J. Ophthalmol..

[19] Sherwin JC (2012). The association between time spent outdoors and myopia using a novel biomarker of outdoor light exposure. Invest. Ophthalmol. Vis. Sci..

[20] Charng J (2019). Estimation of heritability and familial correlation in myopia is not affected by past sun exposure. Ophthalmic Genet..

[21] Dong N (2009). Abnormal epithelial differentiation and tear film alteration in pinguecula. Invest. Ophthalmol. Vis. Sci..

[22] Hogan MJ, Alvarado J (1967). Pterygium and pinguecula: electron microscopic study. Arch. Ophthalmol..

[23] Kanski JJ (2007). Clinical Ophthalmology: A Systematic Approach.

[24] McCarty CA, Lee ES, Livingston PM, Bissinella M, Taylor HR (1996). Ocular exposure to UV-B in sunlight: The Melbourne Visual Impairment Project model. Bull. World Health Organ..

[25] Read SA (2016). Anterior eye tissue morphology: Scleral and conjunctival thickness in children and young adults. Sci. Rep..

[26] Zhang X-R (2013). The effect of age and conjunctivochalasis on conjunctival thickness. Curr. Eye Res..

[27] Sehu KW, Lee WR (2005). Ophthalmic Pathology An Illustrated Guide for Clinicians..

[28] Dhakal R, Vupparaboina KK, Verkicharla PK (2020). Anterior sclera undergoes thinning with increasing degree of myopia. Invest. Ophthalmol. Vis. Sci..

[29] Read SA (2016). Diurnal variation of anterior scleral and conjunctival thickness. Ophthalmic Physiol. Opt..

[30] Nanji AA, Sayyad FE, Galor A, Dubovy S, Karp CL (2015). High-resolution optical coherence tomography as an adjunctive tool in the diagnosis of corneal and conjunctival pathology. Ocul. Surf..

[31] Zhang X-R (2013). Bulbar conjunctival thickness measurements with optical coherence tomography in healthy Chinese subjects. Invest. Ophthalmol. Vis. Sci..

[32] Ebneter A, Häner NU, Zinkernagel MS (2015). Metrics of the normal anterior sclera: Imaging with optical coherence tomography. Graefes Arch. Clin. Exp. Ophthalmol..

[33] Gumus K, Pflugfelder SC (2013). Increasing prevalence and severity of conjunctivochalasis with aging detected by anterior segment optical coherence tomography. Am. J. Ophthalmol..

[34] Abdel-Khalek LM, Williamson J, Lee WR (1978). Morphological changes in the human conjunctival epithelium. I. In the normal elderly population. Br. J. Ophthalmol..

[35] Buckhurst HD, Gilmartin B, Cubbidge RP, Logan NS (2015). Measurement of scleral thickness in humans using anterior segment optical coherent tomography. PLoS ONE.

[36] Osterlind G (1944). An investigation into the presence of lymphatic tissue in the human conjunctiva, and its biological and clinical importance. Acta Ophthalmol..

[37] Schlatter B, Beck M, Frueh BE, Tappeiner C, Zinkernagel M (2015). Evaluation of scleral and corneal thickness in keratoconus patients. J. Cataract Refract. Surg..

[38] Coroneo MT (1993). Pterygium as an early indicator of ultraviolet insolation: A hypothesis. Br. J. Ophthalmol..

[39] Mimura T (2011). Severity and determinants of pinguecula in a hospital-based population. Eye Contact Lens.

[40] Norn MS (1979). Prevalence of pinguecula in Greenland and in Copenhagen, and its relation to pterygium and spheroid degeneration. Acta Ophthalmol..

[41] Utine CA (2009). Autofluorescence imaging of pingueculae. Br. J. Ophthalmol..

[42] Wolffsohn JS, Drew T, Sulley A (2014). Conjunctival UV autofluorescence—Prevalence and risk factors. Cont. Lens Anterior Eye.

[43] Haworth KM, Belair CD (2020). Effect of UV-absorbing contact lenses on conjunctival ultraviolet autofluorescence. Curr. Eye Res..

[44] Ip MH, Chui JJ, Tat L, Coroneo MT (2015). Significance of fuchs flecks in patients with pterygium/pinguecula: Earliest indicator of ultraviolet light damage. Cornea.

[45] Yazar S (2015). Genetic and environmental factors in conjunctival UV autofluorescence. JAMA Ophthal..

